# Lifestyle factors and contact to general practice with respiratory alarm symptoms—a population-based study

**DOI:** 10.1186/s12875-016-0444-9

**Published:** 2016-04-21

**Authors:** Lisa Maria Falk Sele, Sandra Elnegaard, Kirubakaran Balasubramaniam, Jens Søndergaard, Dorte Ejg Jarbøl

**Affiliations:** Research Unit of General Practice, Department of Public Health, University of Southern Denmark, J.B. Winsløws Vej 9A, 5000 Odense C, Denmark

**Keywords:** Lung cancer, Symptoms, Healthcare seeking, Lifestyle factors, General population

## Abstract

**Background:**

A prerequisite for early lung cancer diagnosis is that individuals with respiratory alarm symptoms (RAS) contact a general practitioner (GP). This study aims to determine the proportion of individuals in the general population who contact a GP with RAS and to analyse the association between lifestyle factors and contact to GPs with RAS.

**Methods:**

A web-based survey of 100 000 individuals randomly selected from the Danish Civil Registration System. Items regarding experience of RAS (prolonged coughing, shortness of breath, coughing up blood, and prolonged hoarseness), GP contacts, and lifestyle factors (smoking status, alcohol intake, and body mass index) were included.

**Results:**

In total 49 706 (52.5 %) individuals answered the questionnaire. Overall 7870 reported at least one respiratory alarm symptom, and of those 39.6 % (3 080) had contacted a GP. Regarding specific symptoms, the proportion of individuals that had contacted a GP varied from 27.4 % (prolonged hoarseness) to 47.9 % (shortness of breath). Being a woman and increasing age were significantly associated with a higher proportion of GP contacts. For both genders, current smoking and alcohol intake were significantly associated with lower odds of contacting a GP.

**Conclusion:**

Among individuals with RAS, less than one-half contacted a GP. Gender, age, smoking status, and alcohol intake significantly influenced whether individuals with RAS contacted a GP.

**Trial registration:**

The project has been approved by the Danish Data Protection Agency (journal no. 2011-41-6651).

## Background

Lung cancer is the most common cause of cancer-related death worldwide [1]. Among other factors, stage at diagnosis has been associated with poor survival rates [2, 3]. To diagnose lung cancer at an earlier stage and to optimise the diagnostic process, cancer referral guidelines have been introduced [4, 5]. The lung cancer guidelines define a number of respiratory alarm symptoms (RAS) indicative of lung cancer [6, 7]. Suspicion of lung cancer is most often raised in general practice [8–10] and general practitioners (GPs) are recommended to refer patients reporting RAS for further investigation [6, 7]. However, one prerequisite for GPs to be able to refer patients with RAS is that individuals with RAS contact a GP.

Current knowledge about the interval from the first symptom experience to GP contact is based mainly on retrospective studies conducted among selected groups of patients already diagnosed with lung cancer, and may thus be flawed by recall bias. The literature points to the fact that RAS arise long before presentation to the GP [11, 12]. Multiple factors may affect people’s decisions about healthcare seeking [13, 14]. Little is, however, known about individuals with RAS and contact to general practice.

Factors that might affect the decision to contact a GP with RAS include socioeconomic status, experience with illness, and lifestyle factors (*e.g.*, smoking status, alcohol intake, and body mass index (BMI)). Both smoking and alcohol intake have been associated with a prolonged interval from symptom experience to diagnosis in head and neck cancers [15], and smoking in particular has been highlighted as a reason for having postponed GP contact among patients with lung cancer [16, 17]. An enhanced understanding of healthcare seeking behaviour in the general population, may improve policy interventions targeting health care seeking with RAS in different groups.

The objectives of this study were to determine the proportion of individuals in the general Danish population, who contact a GP with RAS and to analyse the association between lifestyle factors and contact to GPs with RAS.

## Methods

### Study design and population

The study was designed as a nationwide cohort study of 100 000 adults aged 20 years or older, randomly selected from the general population. The project was approved by the Danish Health Authority, who provided a random sample from the Danish Civil Registration System (CRS), where all Danish citizens are registered with a unique personal identification number. The CRS contains information on every Danish resident’s date of birth, gender, migration, *etc.* [18]. Each invited individual received a postal letter explaining the purpose of the study. The letter also contained a unique 12-digit login for a secure webpage. This provided access to a web-based questionnaire about health, symptoms, and GP contacts. Individuals without access to a computer, tablet, or smartphone were offered the opportunity to complete the survey as a telephone interview.

### The questionnaire

A comprehensive questionnaire concerning the experience of 44 predefined specific and nonspecific cancer alarm symptoms, as well as general and frequent symptoms, was developed. The alarm symptoms were selected based on a review of literature including national and international cancer referral guidelines [6, 7, 10, 19]. This paper focus on four RAS: prolonged coughing, shortness of breath, coughing up blood, and prolonged hoarseness [7]. The questionnaire was based on standard rating scales, previously validated questionnaires, and ad hoc items. The methodological framework for developing, pilot testing, and field testing the questionnaire is described in detail elsewhere [20].

The respondents were asked whether they had experienced one or more of the symptoms within the preceding four weeks, and whether they had contacted a GP about the symptom(s). The wording of the question regarding symptoms was: “Have you experienced any of the following bodily sensations, symptoms, or discomforts within the past four weeks? (Tick off yes for each positive answer)” The question regarding contacting a GP was: “Have you contacted your GP concerning the symptom(s) you have experienced within the preceding four weeks, through appointment, by telephone, or email? (Yes/no)”.

An item concerning when the symptom(s) first occurred was also included. The response categories were: “Less than one month ago”, “1–3 months ago”, “3–6 months ago” or “more than six months ago”. Questions regarding current smoking status, average alcohol intake, weight, and height were also asked.

### Statistical analyses

In the lung cancer referral guideline, coughing and hoarseness are defined as alarm symptoms when prolonged, i.e., when lasting for more than 4 to 6 weeks and 3 to 4 weeks, respectively [7]. To comply with these definitions, only respondents who first experienced the symptom more than one month ago were considered to have experienced prolonged coughing and prolonged hoarseness.

Covariates considered in the uni- and multivariate statistical analyses were gender, age, smoking status, alcohol intake, and BMI. The respondents were divided into the following age groups: 20–39, 40–59, 60–79, and ≥80 years. Smoking status was categorised as current, former, and never smokers. Alcohol intake was categorised according to units per week: 0, 1–7, 8–21, and ≥ 22 units/week. BMI was calculated from height and weight, and categorised according to the World Health Organization guidelines [21]: underweight (BMI <18.5), normal weight (BMI 18.5–24.9), overweight (BMI 25–29.9), and obese (BMI ≥30).

We calculated the proportion of individuals who contacted a GP with at least one of the RAS, as well as the proportion of individuals who contacted a GP with each of the RAS. Confidence intervals were calculated using the binomial distribution. Differences between the proportion of individuals with RAS that contacted a GP and each covariate were tested with either the chi-squared test or Fisher’s exact test, as appropriate.

Multivariate logistic regression models were used to analyse associations between lifestyle factors and contact to GP with RAS. Adjustments were made for possible confounders: age, smoking status, alcohol intake, and BMI [22, 23]. To evaluate collinearity between lifestyle factors, correlation coefficients were calculated with Spearman’s rank correlation.

Logistic regression was used to test for interaction between gender and each covariate for contact to GP with at least one respiratory alarm symptom, prolonged coughing, shortness of breath, or prolonged hoarseness, respectively. The tests were made with Bonferroni adjustment to account for multiple testing. Tests for interaction and multivariate logistic regression models were not made for coughing up blood because of the small number of respondents with this symptom who contacted a GP. As a result of statistical interactions, the multivariate analyses were stratified with respect to gender.

Linear trends in contacting a GP to report RAS were tested with logistic regression models for age and alcohol intake. All analyses were repeated with the subgroup of people aged 40 and older, owing to the higher risk of lung cancer in this age group.

Statistical tests were made using significance threshold 0.05. Data analyses were conducted using Stata IC 13©.

## Results

**Fig. 1:**
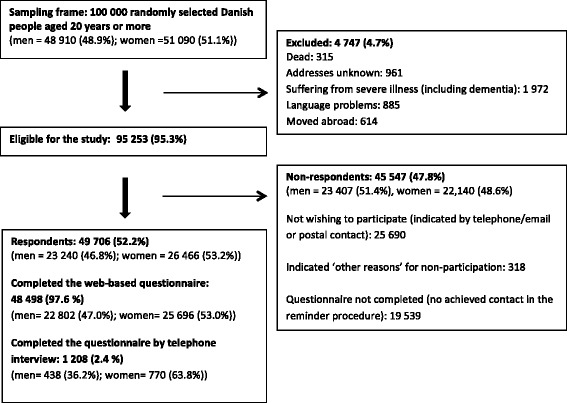
Study cohort

Of the 100 000 invited individuals, 4 747 (4.7 %) were ineligible because they had died, could not be reached (address unknown), were suffering from severe illnesses (including dementia), had language problems, or had moved abroad. Of the 95 253 (95.3 %) eligible individuals, 49 706 individuals completed the questionnaire, yielding an overall response rate of 52.2 % (Fig. 1). The median age of the respondents was 52 years (interquartile range (IQR) 40–64) compared to 50 years (IQR 36–66) for non-respondents. Slightly more respondents were women (53.2 %). Table 1 lists the descriptive data of the respondents.

**Table 1:** Characteristics of the respondents

	Number	Percent
Total	49 706	100.0
Gender		
Men	23 240	46.8
Women	26 466	53.2
Age		
20–39	12 251	24.6
40–59	20 305	40.9
60–79	15 748	31.7
> 80	1 402	2.8
Smoking status*		
Never	21 895	45.9
Former	15 529	32.5
Current	2 082	21.6
Alcohol intake*		
0 units/week	3 668	7.7
1–7 units/week	23 321	66.3
8–21 units/week	10 708	22.4
≥ 22 units/week	1 729	3.6
Body Mass Index*		
Underweight (<18.5)	756	1.6
Normal (18.5–24.9)	23 168	48.7
Overweight (25–29.9)	16 668	35.0
Obese (≥30)	6 990	14.7

*Total numbers for each group may not add to full sample due to missings

Tables 2 and 3 depict the proportion of individuals who contacted a GP with at least one respiratory alarm symptom and with each individual respiratory alarm symptom, respectively. Of those reporting at least one respiratory alarm symptom, 39.6 % had contacted a GP (Table 2). The greatest proportion of contact to a GP were found for shortness of breath (49.7 %), and the smallest proportion of contact to a GP were found for prolonged hoarseness (27.4 %) (Table 3).

**Table 2:** Proportion of contacts to GP with at least one respiratory alarm symptom by gender, age, smoking status, alcohol intake and body mass index

	Symptom experience	Contact to GP			
	*n*	*n*	%*	95 % CI	*P*-value^a^
Total	7 870	3 080	39.6	38.5–40.7	
Gender					0.047
Men	3 978	1 483	38.5	37.7–40.1	
Women	3 892	1 597	40.7	39.2–42.3	
Age					<0.001
20–39	1 643	435	26.7	24.6–29.0	
40–59	2 794	989	35.6	33.8–37.4	
60–79	3 044	1 462	48.7	46.9–50.3	
> 80	389	194	52.7	47.5–50.5	
Smoking status					<0.001
Never	2 480	979	39.5	37.5–41.4	
Former	2 488	1 196	48.1	46.1–50.1	
Current	2 519	788	31.3	29.5–33.1	
Alcohol intake					<0.001
0 units/week	813	383	47.1	43.6–50.6	
1–7 units/week	3 114	1750	39.5	38.0–40.9	
8–21 units/week	1 828	685	37.5	35.2–39.7	
≥ 22 units/week	409	144	35.2	30.6–40.1	
Body Mass Index					<0.001
Underweight (<18.5)	154	64	41.6	33.7–49.8	
Normal (18.5–24.9)	3 214	1 201	37.4	35.7–39.1	
Overweight (25–29.9)	2 644	1 048	39.6	37.8–41.5	
Obese (≥30)	1 435	631	44.0	41.4–46.6	

^a^Tested for difference between groups with chi-square test

*Percentages might not fully match to total numbers due to missings. Missings do not exceed 1 %

**Table 3:** Proportions of contact to GP with respiratory alarm symptoms by gender, age, smoking status, alcohol intake and BMI

	Prolonged coughing					Shortness of breath					Coughing up blood					Prolonged hoarseness				
	Symptom experience	Contact to GP				Symptom experience	Contact to GP				Symptom experience	Contact to GP				Symptom experience	Contact to GP			
	*n*	*n*	%*	95 % CI	*P*-value^a^	*n*	*n*	%*	95 % CI	*P*-value^a^	*n*	*n*	%*	95 % CI	*P*-value^b^	*n*	*n*	%*	95 % CI	*P*-value^a^
Total	4 180	1 438	34.7	33.2–36.7		3 960	1 936	49.7	48.1–51.3		62	29	47.5	34.6–60.7		1 694	458	27.4	25.2–29.6	
Gender					<0.001					<0.001					0.586					0.274
Men	2 095	658	31.7	29.7–33.8		1 912	960	50.9	48.6–53.2		42	18	43.9	28.5–60.3		813	210	26.1	23.1–29.3	
Women	2 085	780	37.7	35.6–39.8		2 048	976	48.5	46.3–50.7		20	11	55.0	31.5–76.9		881	248	28.5	25.5–31.6	
Age					<0.001					<0.001					0.295					<0.001
20–39	813	184	22.9	20.0–25.9		880	288	33.1	29.9–36.3		18	8	44.4	21.5–69.2		303	32	10.7	7.4–14.7	
40–59	1 452	435	30.0	27.6–32.4		1 423	632	44.8	42.2–47.5		23	8	36.4	17.2–59.3		519	136	26.4	22.6–30.4	
60–79	1 696	723	43.0	40.7–45.4		1 457	883	61.8	59.2–64.3		23	12	60.0	36.1–80.1		750	251	34.1	30.7–37.7	
> 80	219	96	45.7	38.9–52.7		200	133	71.1	64.1–77.5		1**	1	100.0	36.1–80.1		122	39	32.0	23.8–41.0	
Smoking status					<0.001					<0.001					0.340					<0.001
Never	1 262	482	38.2	35.5–40.9		1 224	562	45.9	43.1–48.8		19	7	36.8	16.3–61.6		567	165	29.1	25.4–33.0	
Former	1 097	471	42.9	40.0–45.9		1 387	809	58.3	55.7–60.9		20	12	60.0	36.1–80.9		617	193	31.3	27.6–35.1	
Current	1 635	428	26.2	24.1–28.4		1 131	490	43.3	40.4–46.3		18	10	55.6	30.8–78.5		430	88	20.5	16.7–24.6	
Alcohol intake					<0.001					0.142					0.281					0.239
0 units/week	409	175	42.8	37.9–47.7		489	267	45.6	50.1–59.1		7	5	71.4	23.9–96.3		167	53	31.7	24.8–39.4	
1–7 units/week	1 642	806	35.2	33.2–37.2		1 510	1 105	48.9	46.9–51.0		25	19	55.9	37.9–72.8		706	282	28.4	25.6–31.3	
8–21 units/week	1 043	329	31.5	28.7–34.5		820	401	48.9	45.4–52.4		13	4	30.8	9.1–61.4		375	90	24.0	19.8–28.7	
≥ 22 units/week	251	70	27.9	22.4–33.9		174	87	50.0	42.3–57.7		3**	1	33.3	8.4–90.1		79	21	26.6	17.3–37.8	
BMI					0.007					0.838					0.911					0.139
Underweight (<18.5)	88	36	40.9	30.5–51.9		79	41	51.9	40.4–63.3		1**	1	100.0	2.5–1		43	13	30.2	17.2–46.1	
Normal (18.5–24.9)	1 731	570	32.9	30.7–35.2		1 494	732	49.0	46.4–51.6		25	12	48.0	27.8–65.7		734	183	24.9	21.8–28.2	
Overweight (25–29.9)	1 409	474	33.6	31.2–36.2		1 297	642	49.9	46.7–52.2		19	9	47.4	24.4–71.1		556	162	29.1	25.4–33.1	
Obese (≥30)	737	291	39.5	35.9–43.1		855	434	50.7	47.4–52.2		12	7	58.3	27.7–84.8		273	86	31.5	26.0–37.4	

^a^Tested for difference between groups with chi-square test

^b^Tested for difference between groups with Fischer’s exact test

*Percentages might not fully match to total numbers due to missings. Missings do not exceed 1 %

**Insufficient variation in this category

A significantly higher proportion of women had contacted a GP with at least one respiratory alarm symptom (*p* = 0.047) (Table 2). The difference between genders persisted when analysing prolonged coughing and shortness of breath separately (Table 3). The proportion of individuals who had contacted a GP differed significantly with regard to age group (*p* < 0.001) and smoking status (*p* < 0.001) for all RAS except coughing up blood (Tables 2 and 3). The proportion of individuals who reported GP contact was higher among people in the oldest age groups for all RAS except prolonged hoarseness. Former and never smokers reported higher proportion of GP contact for three of the four RAS when compared to current smokers. No significant differences were found between GP contact, BMI and alcohol intake except for individuals reporting prolonged coughing.

Tables 4 and 5 demonstrate the associations between age, lifestyle factors, and contacting a GP with at least one respiratory alarm symptom, prolonged coughing, shortness of breath and prolonged hoarseness, for men and women, respectively. Due to Interactions between gender and some covariates regarding contact to GP with RAS the analyses were carried out separately for men and women. Analyses regarding coughing up blood were not possible due to few GP contacts. Lifestyle factors were included in the same multivariate logistic regression model because of low correlation coefficients (data not shown).

**Table 4:** Associations between lifestyle factors and contact to GP with respiratory alarm symptoms for men

	At least one respiratory alarm symptom				Prolonged coughing				Shortness of breath				Prolonged hoarseness			
	OR	Adj. OR^a^	95 % CI	*P*-value^c^	OR	Adj. OR^a^	95 % CI	*P*-value^c^	OR	Adj. OR^a^	95 % CI	*P*-value^c^	OR	Adj. OR^a^	95 % CI	*P*-value^c^
Age				<0.001				<0.001				<0.001				<0.001
20–39	1	1			1	1			1	1			1	1		
40–59	**1.57**	**1.45**	1.18–1.78		**1.54**	**1.52**	1.12–2.07		**1.80**	**1.71**	1.30–2.25		**2.38**	**2.00**	1.03–3.86	
60–79	**2.75**	**2.41**	1.96–2.95		**2.81**	**2.64**	1.96–3.55		**3.50**	**3.19**	2.40–4.24		**4.12**	**3.17**	1.07–5.89	
> 80	**3.41**	**2.94**	2.05–4.22		**3.16**	**3.06**	1.90–4.92		**7.63**	**6.02**	3.33–10.89		**4.49**	**3.71**	1.64–8.37	
Smoking status																
Never	1	1			1	1			1	1			1	1		
Former	**1.52**	**1.19**	1.00–1.42		1.26	0.98	0.76–1.27		**1.90**	**1.36**	1.04–1.73		1.45	1.13	0.77–1.68	
Current	**0.62**	**0.59**	0.49–0.71		**0.53**	**0.50**	0.39–0.64		**0.78**	**0.67**	0.51–0.86		**0.61**	0.65	0.40–1.06	
Alcohol intake				0.020^b^				0.040^b^				0.103^b^				0.892^b^
0 units/week	1	1			1	1			1	1			1	1		
1–7 units/week	**0.68**	**0.63**	0.49–0.71		0.78	**0.69**	0.47–1.00		**0.64**	**0.61**	0.43–0.87		1.2	1.07	0.56–2.07	
8–21 units/week	**0.69**	**0.63**	0.49–0.82		0.76	0.68	0.46–1.00		**0.70**	**0.60**	0.41–0.87		1.12	1.03	0.52–2.04	
≥ 22 units/week	**0.63**	**0.59**	0.48–0.82		**0.61**	**0.54**	0.33–0.87		0.68	**0.62**	0.39–1.00		1.19	1.15	0.50–2.67	
Body Mass Index																
Underweight (<18.5)	0.88	0.98	0.46–2.90		1.84	2.41	0.96–6.01		0.75	0.72	0.20–4.62		0.33	0.46	0.05–3.83	
Normal (18.5–24.9)	1	1			1	1			1	1			1	1		
Overweight (25–29.9)	1.07	0.98	0.84–1.15		1.09	1.00	0.80–1.24		0.87	**0.76**	0.61–0.96		**1.46**	1.29	0.90–1.87	
Obese (≥30)	**1.38**	1.21	1.00–1.46		**1.52**	**1.36**	1.03–1.79		0.95	**0.75**	0.58–0.98		1.18	1.10	0.67–1.79	

*Adj* Adjusted

^a^Adjusted for all variables: age, smoking status, alcohol intake and body mass index (except when the variable itself was being examined)

^b^Only tested for those who drink more than 0 units/week

^c^Test for trend

Bold indicates significance at 5 % level

**Table 5:** Associations between lifestyle factors and contact to GP with respiratory alarm symptoms for women

	At least one respiratory alarm symptom				Prolonged coughing				Shortness of breath				Prolonged hoarseness			
	OR	Adj. OR^a^	95 % CI	*P*-value^c^	OR	Adj. OR^a^	95 % CI	*P*-value^c^	OR	Adj. OR^a^	95 % CI	*P*-value^c^	OR	Adj. OR^a^	95 % CI	*P*-value^c^
Age				<0.001				<0.001				<0.001				<0.001
20–39	1	1			1	1			1	1			1	1		
40–59	**1.48**	**1.47**	1.21–1.77		**1.33**	**1.4**	1.05–1.87		**1.52**	**1.49**	1.17–1.91		**3.51**	**3.41**	1.95–5.98	
60–79	**2.51**	**2.52**	2.08–3.04		**2.23**	**2.33**	1.76–3.08		**3.09**	**3.31**	2.56–4.28		**4.61**	**4.74**	2.73–8.23	
> 80	**2.78**	**2.37**	1.70–3.32		**2.58**	**2.13**	1.31–3.46		**3.70**	**3.26**	2.05–5.18		**3.52**	**3.45**	1.62–7.37	
Smoking status																
Never	1	1			1	1			1	1			1	1		
Former	**1.33**	**1.20**	1.02–1.41		1.23	1.14	0.90–1.45		**1.39**	1.17	0.94–1.47		0.89	0.78	0.54–1.13	
Current	**0.79**	**0.83**	0.70–0.98		**0.64**	**0.67**	0.53–0.83		1.03	1.05	0.84–1.33		**0.66**	0.73	0.49–1.09	
Alcohol intake				<0.001^b^				<0.001^b^				0.004^b^				0.016^b^
0 units/week	1	1			1	1			1	1			1	1		
1–7 units/week	**0.77**	**0.72**	0.59–0.88		0.72	**0.67**	0.50–0.88		0.88	0.83	0.64–1.06		0.73	0.70	0.45–1.10	
8–21 units/week	**0.64**	**0.57**	0.44–0.72		**0.55**	**0.51**	0.36–0.71		0.75	**0.60**	0.43–0.83		0.48	**0.46**	0.26–0.83	
≥ 22 units/week	**0.53**	**0.48**	0.28–0.80		0.59	0.54	0.28–1.03		0.80	0.72	0.31–1.64		0.59	0.59	0.17–2.01	
Body Mass Index																
Underweight (<18.5)	1.26	1.19	0.81–1.74		1.19	1.13	0.67–1.89		1.29	1.05	0.63–1.76		1.68	1.76	0.80–3.86	
Normal (18.5–24.9)	1	1			1	1			1	1			1	1		
Overweight (25–29.9)	**1.17**	1.03	0.88–1.21		1.06	0.92	0.74–1.15		1.15	0.99	0.79–1.24		1.04	0.89	0.61–1.29	
Obese (≥30)	**1.26**	1.11	0.93–1.33		1.20	1.07	0.83–1.38		1.16	0.99	0.78–1.26		**1.59**	1.45	0.96–2.21	

*Adj* Adjusted

^a^Adjusted for all variables: age, smoking status, alcohol intake and body mass index (except when the variable itself was being examined)

^b^Only tested for those who drink more than 0 units/week

^c^Test for trend

Bold indicates significance at 5 % level

### Age

Among both men and women, a significant trend between increasing age and being more likely to contact a GP with at least one respiratory alarm symptom (P_trend_ < 0.001), as well as each respiratory alarm symptom (P_trend_ < 0.001) was observed (Tables 4 and 5).

### Smoking status

Former smoking was a statistically significant determinant for contacting a GP with at least one respiratory alarm symptom for both men (OR = 1.19, 95 % CI 1.00–1.42) and women (OR = 1.20, 95 % CI 1.02–1.41). Current smokers had significantly lower odds of contacting a GP with at least one respiratory alarm symptom among both men (OR = 0.59, 95 % CI 0.49–0.71) and women (OR = 0.83, 95 % CI 0.70–0.98). This was also the case for both genders with prolonged coughing (OR_men_ = 0.50 95 % CI 0.39–0.64; OR_women_ = 0.67, 95 % CI 0.53–0.83), and among men with shortness of breath (OR = 0.67, 95 % CI 0.51–0.86) (Tables 4 and 5).

### Alcohol intake

Increasing alcohol intake was associated with decreasing odds of contacting a GP with at least one respiratory alarm symptom for both men (P_trend_ = 0.02) and women (P_trend_ < 0.001). The tendency persisted when analysing men with prolonged coughing (P_trend_ = 0.04) and when analysing women with prolonged coughing (P_trend_ < 0.001), shortness of breath (P_trend_ = 0.004), and prolonged hoarseness (P_trend_ = 0.016), respectively (Tables 4 and 5).

### Body mass index

Obese men with prolonged coughing were more likely to contact a GP (OR = 1.36, 95 % CI 1.03–1.79) than men with normal weight. Among men with shortness of breath, overweight (OR = 0.76, 95 % CI 0.61–0.96) and obesity (OR = 0.75, 95 % CI 0.58–0.98) were significantly associated with not opting to contact a GP (Table 4). Among women, no statistically significant associations were observed between BMI and contacting a GP (Table 5).

When analysing the subgroup of people aged 40 years or older, all tendencies and associations persisted for both men and women (data not shown).

## Discussion

### Summary of main findings

In this nationwide study comprising 49 706 individuals from the general population, 39.6 % of those reporting RAS had contacted a GP with at least one of the symptoms. The proportion of GP contacts ranged from 27.4 % (prolonged hoarseness) to 49.7 % (shortness of breath). In general, more women than men had contacted a GP with RAS, and the proportion of individuals that contacted a GP increased with increasing age.

Contacting a GP with RAS was significantly associated with lifestyle factors. Current smokers were less likely to contact a GP than never smokers, and the odds of contacting a GP decreased with increasing alcohol intake.

### Discussion of results and comparison with existing literature

Respiratory symptoms are frequently presented in general practice [24], but few studies have estimated the proportion of individuals in the general population who contact a GP with RAS [25–27]. In a population of individuals younger than 60 years, Elliot et al. found that 7.0 % of individuals with coughing and 18.2 % of individuals with shortness of breath contacted a GP [25], proportions much lower than in the present study. Whitaker et al. found that 55.7 % of individuals older than 50 years with persistent cough or hoarseness contacted a GP [26], and Svendsen et al. found that 69.4 % of people older than 40 years with coughing for more than 6 weeks contacted a GP [27], rates that are substantially higher than in the present study*.* Differences in age groups and time frames might explain some of these differences.

Overall women were more likely to contact general practice about RAS compared to men. The same tendency has been shown for coughing in a previous Danish study [27]. Looking at single symptoms the tendency did however not apply for shortness of breath, where men were more likely to contact a GP. Possible explanations for the gender difference could be to which degree the symptoms interfere with daily life activity or how worrying men and women, respectively, find the symptoms. Women might in general be more aware about the symptoms leading to more contacts to general practice [28]. Men might, however, find that shortness of breath interfere more with their daily living, resulting in more contacts to general practice. These hypotheses will be tested in future studies.

The proportion of individuals with RAS that contacted a GP varied between 27.4 % (prolonged hoarseness) and 49.7 % (shortness of breath). This finding indicates that symptoms defined as cancer alarm symptoms in general practice are not necessarily interpreted as alarming in the general population, but instead registered as a part of everyday life. The positive predictive values of RAS presented in general practice are low [29], entailing that many contacts are required to find the individuals who will actually be diagnosed with lung cancer. In order to decrease the time from first symptom presentation to contact with a GP, additional knowledge and understanding of reasons for healthcare-seeking behaviour are needed.

Risk of lung cancer increases with age, and individuals older than 40 years, especially smokers, are at higher risk of developing lung cancer than younger individuals [7, 30]. Furthermore, age is undoubtedly an important factor in the evaluation of patients’ symptom presentation in general practice, and decisions about referral for further investigation will often depend on the patient’s age. In the present study, the proportion of individuals that contacted a GP because of RAS increased with age, which is in line with the findings of a previous Danish study [27]. To determine the influence of lifestyle factors on establishing GP contact in the age group with increased risk of lung cancer, we analysed the associations for individuals aged 40 years or older. This did, however, not change the associations with lifestyle factors notably.

Current smoking was negatively associated with contacting a GP with RAS. These findings are supported by Smith et al., who found that smoking status was associated with a prolonged interval between the first symptom experience and contacting a GP [13]. The negative association might be due to current smokers interpreting their symptoms as normal [13], or not realising that they have an increased risk of disease [31]. Current smokers might also feel ashamed or fear being stigmatised [12, 32], and therefore opt not to contact a GP.

In the present study, individuals reporting an alcohol intake were less likely to contact a GP to report RAS, and the odds of a person with RAS would contact a GP decreased with increasing alcohol intake. One possible explanation for this is that individuals with no reported alcohol intake have deliberately chosen healthy living and therefore take action when they experience a symptom. Another explanation might be that individuals reporting an alcohol intake are more willing to take a risk than individuals reporting no alcohol intake [33]. These suggestions are hypothetical and should be examined in detail in future studies.

RAS are defined as warnings signs of cancer, but obviously not all symptoms lead to referral for further examination, prescription of medicine or malignant diagnoses. Using a unique personal identification number assigned to all Danish citizens prospective data from the Danish national registers regarding referral to hospital, lung cancer diagnoses and use of medication will be linked to data from the questionnaire. This will enhance the understanding of the significance of RAS in the general population and will provide knowledge about their predictive value for subsequent disease.

### Strengths and limitations

A strength of this study is the large study sample of 100 000 individuals randomly selected from the general Danish population. A total of 52.2 % answered the questionnaire. The response rate was higher than in two similar population-based studies from the United Kingdom [25, 26], but lower than in a previous Danish study [27]. Although a preponderance of the respondents were women and the respondents were slightly older than the non-respondents, the respondents were fairly representative of the general Danish population. However, differences between the respondents and the non-respondents regarding other parameters, which might include a risk of over- or underestimating the proportion of GP contacts, cannot be eliminated. For more details see Elnegaard et al. [34]. Furthermore it should be acknowledged that despite the large number of participants, the study was unable to detect possible differences between subgroups in regard to *e.g.* contact to GP with coughing up blood. An even larger sample might have enhanced the statistical power.

Prevalence estimates of symptoms and the proportion of GP contacts might be overestimated if the willingness to complete the questionnaire has been affected by the presence of many RAS or frequent contact to a GP [35, 36]. On the other hand, individuals with many symptoms or GP contacts might not have the surplus energy to complete a questionnaire, which would counterbalance the above-mentioned.

The fact that the questionnaire was web-based might result in selection of the respondents, *e.g.* exclusion of the elderly. To reduce potential selection bias, individuals without access to a computer, smartphone, or tablet were offered the opportunity to conduct the survey as a telephone interview. Nevertheless, a lower response rate was observed in the oldest age group. This finding might indicate that the telephone interview has not completely compensated for the possible selection. The lower response rate might result in bias, as respondents might be in better health than non-respondents. Thus, the proportion of individuals that contacted a GP among the oldest age group might be even higher than estimated in the present study.

The study participants were asked to recall symptom experiences within the preceding four weeks and whether they at any time had contacted a GP with these symptoms. Recall bias cannot be completely eliminated in questionnaire studies [37]. Some may misplace older symptom experiences in the specified timeframe due to the severity of symptoms or because they had contacted a GP about them [38]. Others may have forgotten about a symptom experience or GP contact, because the symptom turned out to be nothing to worry about or simply due to memory decay [39]. The recall period was chosen because it was found reasonable to assume that people were able to recall symptom experiences and GP contacts fairly accurately within this timespan [40, 41].

A general tendency to underreport smoking status, alcohol intake, and weight, and to overreport height, could introduce bias to the self-reported lifestyle factors [42–44]. However, web-based questionnaires have been suggested to enhance the perception of privacy among participants, increasing the reliability of responses regarding sensitive subjects such as lifestyle factors [45, 46]. Although misclassification cannot be eliminated, the questionnaire was comprehensive and considered a wide range of different symptom experiences and items. Thus, it is unlikely that, for example, questions asked at the beginning of the questionnaire about experience of RAS or contacting a GP about RAS have significantly affected later responses regarding lifestyle factors. A possible misclassification would therefore be non-differentiated [37].

Misunderstanding and misinterpretation of the items are well known weaknesses in questionnaire-based studies. To minimise the risk of misinterpretation, the conceptual framework and wording of the questions were discussed with representatives from three different disciplines: psychology, anthropology, and medical science, prior to the final questionnaire. Furthermore, several pilot tests and a pilot study were conducted to enhance the comprehensibility of the questionnaire [20].

To account for possible confounders, the analyses were adjusted for age, smoking status, alcohol intake, and BMI [22, 23, 47]. We also considered whether other demographic factors could be confounders. Literature regarding demographic factors, healthcare seeking, and the interval between the first symptom experience and contacting a GP has observed few significant associations, but with ambiguous and inconclusive tendencies [14, 25, 27, 48]. Thus, demographic factors were not included in the multivariate analyses.

## Conclusion and implications

Among individuals reporting RAS, 39.6 % had contacted a GP. The proportion of individuals who contacted a GP was highest for shortness of breath and lowest for prolonged hoarseness. More women reporting RAS contacted a GP than men, and the proportion that contacted a GP increased with age. Lifestyle factors were significantly associated with contacting a GP with RAS. Current smokers were less likely to contact a GP, and the likelihood of contacting a GP with RAS decreased with increasing alcohol intake.

This study contributes with important estimates of the proportion of individuals in the general population and among different subgroups, who contacted a GP with RAS. A special focus on smokers with RAS who do not contact a GP could lead to earlier presentation and subsequent referral, thereby increasing the chance of diagnosing lung cancer at an earlier stage. One option might be targeting lung cancer awareness campaigns towards current smokers. Other possibilities include increasing the approachability of GPs by encouraging current smokers to initiate contact if they experience RAS [49], and GPs asking about RAS when smokers contact them for other reasons.

Future research should explore considerations ahead of GP contacts and reasons for not contacting a GP when experiencing RAS in the general population and among subgroups. This information might increase understanding of the interval between the first symptom experience and contacting a GP, which might help optimise communication between GPs and patients and contribute to achieving appropriate healthcare seeking among people with cancer alarm symptoms.

### Ethical approval

The Regional Scientific Ethics Committee for Southern Denmark evaluated the project and concluded that the project was not notifiable and could be implemented according to Danish legislation. All participants consented to participate in the study. The participants were clearly informed that there would be no clinical follow-up, and that they should contact their own GP in case of concern or worry. The project has been approved by the Danish Data Protection Agency (journal no. 2011-41-6651).

### Availability of data and materials

Due to data protection regulations of the Danish Data Protection, Statistics Denmark and the Danish Health and Medicines Authority, the access to data is strictly limited to the researchers who have obtained permission for data processing.

## Abbreviations

BMI: body mass index
CRS: Danish Civil Registration System
GP: general practitioner
RAS: respiratory alarm symptoms
